# Tackling tack removal after use of the through-the-scope helix tack and suture device for stent fixation

**DOI:** 10.1055/a-2094-9601

**Published:** 2023-06-15

**Authors:** Natalie Wilson, Mohamed Abdallah, Eric M. Pauli, Mohammad Bilal

**Affiliations:** 1 Department of Internal Medicine, University of Minnesota Medical Center, Minneapolis, Minnesota, USA; 2 Division of Gastroenterology and Hepatology, University of Minnesota Medical Center, Minneapolis, Minnesota, USA; 3 Division of Minimally Invasive and Bariatric Surgery, Department of Surgery, Penn State Health Milton S. Hershey Medical Center, Hershey, Pennsylvania; 4 Division of Gastroenterology and Hepatology, Minneapolis Veterans Affairs Health Care System, Minneapolis, Minnesota, USA

The through-the scope (TTS) helix tack-and-suture device (X-Tack; Apollo Endosurgery, Austin, Texas, USA) is a newer endoscopic suturing device used for closure of large defects after endoscopic resection 1 2 . This device has recently been gaining popularity as a tool for stent fixation 1 3 4 . One of the limitations of this device for stent fixation is the lack of a dedicated removal tool. Here, we demonstrate two options for helical tack removal following application for stent fixation.

A 74-year-old man with a benign duodenal peptic stricture presented for removal of a previously placed fully covered lumen-apposing metal stent that had been fixated using the TTS tack and suture device in stent-mucosa-mucosa-stent fashion. An upper endoscopy was performed and showed the previously placed stent in the first portion of the duodenum with four helical tacks and suture in place. The suture was cut using endoscopic scissors (Ensizor; Slater Endoscopy, Miramar, Florida, USA) followed by tack removal using a TTS rotatable endoclip (Resolution 360; Boston Scientific, Marlborough, Massachusetts, USA) and a rotatable rat tooth forceps (Raptor 360; Steris, Mentor, Ohio, USA) ( Video 1 ). The stent was then successfully removed. No adverse events occurred.

**Video 1** Approaches to helical tack removal in a patient who underwent stent fixation with the X-tack device.


The TTS tack-and-suture device was initially designed as a mucosal-based tissue closure device but more recently has been used for endoscopic device fixation 1 3 4 . One of the limitations for its use is the lack of a dedicated removal device, which can hamper stent removal and complicate future interventions where tack removal is necessary (e. g., reclosing a defect in the event of suture breakage around the tacks). First described by Pauli, endoscopic grasping devices capable of rotating in an anti-clockwise direction can be utilized to hold and unscrew helical tacks 5 . In this case, the rotatable endoclip and rotatable rat tooth forceps were used to remove the tacks. It is also important to note that when the stent was initially placed, a degree of laxity on the suture was intentionally maintained to facilitate cutting of the suture for subsequent stent removal. These methods may serve as a useful insight for endoscopists when using the TTS tack-and-suture device for stent fixation or when a need arises to remove tacks for other indications.

Endoscopy_UCTN_Code_TTT_1AR_2AG

## Notes

Endoscopy E-Videos

https://eref.thieme.de/e-videos
*E-Videos* is an open access online section of the journal *Endoscopy* , reporting on interesting cases and new techniques in gastroenterological endoscopy. All papers include a high-quality video and are published with a Creative Commons CC-BY license. Endoscopy E-Videos qualify for HINARI discounts and waivers and eligibility is automatically checked during the submission process. We grant 100% waivers to articles whose corresponding authors are based in Group A countries and 50% waivers to those who are based in Group B countries as classified by Research4Life (see: https://www.research4life.org/access/eligibility/ ). This section has its own submission website at https://mc.manuscriptcentral.com/e-videos
